# Government policy targeting antimicrobial use in production animals: A call to action commentary

**DOI:** 10.1016/j.onehlt.2025.101221

**Published:** 2026-06-10

**Authors:** Fiona Emdin, Kayla Strong, Daniela Corno, Jaskeerat Singh, Susan Rogers Van Katwyk, Heather Ganshorn, Arne Ruckert, Jeremy M. Grimshaw, Mathieu JP Poirier

**Affiliations:** a Global Strategy Lab, Dahdaleh Institute for Global Health Research, Faculty of Health and Osgoode Hall Law School, York University, 4700 Keele Street, Dahdaleh Building 2120, Toronto, Ontario M3J 1P3, Canada; b Libraries and Cultural Resources, University of Calgary, Calgary, Alberta, Canada; c Methodology and Implementation Research Program, Ottawa Hospital Research Institute, Ottawa, ON, Canada; d Department of Medicine, University of Ottawa, ON, Canada

**Keywords:** AMR, Antimicrobial resistance, Policy, Government, Systematic review, Decision making

## Abstract

This commentary is submitted in support of a systematic review titled “Government policy interventions to reduce veterinary antimicrobial consumption in production animals: a systematic review and evidence map,” which is currently under review for publication. While the systematic review synthesizes and critically appraises the global evidence on government policy interventions to reduce antimicrobial use (AMU) and antimicrobial resistance (AMR) in production animals, this commentary highlights the key policy implications of that evidence.

Driven in part by antimicrobial use, antimicrobial resistance (AMR) is a burgeoning public health threat that contributed to almost 5 million human deaths in 2021 [1]. Two-thirds of all antimicrobials are used in production animals, a term used to mean any species of animal raised to produce products for consumption, such as dairy cows or meat chickens [2]. In response, governments around the world have implemented policies to improve responsible antimicrobial use and address antimicrobial resistance in production animals.

Governments need evidence to guide effective policy interventions, understood as any initiative used by the government to shape policies, sectors or society [3]. To fill this knowledge gap, we recently completed a systematic review and evidence map examining government policy interventions that target veterinary antimicrobial use or resistance in production animals [4]. Of over 30,000 studies screened, we identified 40 that met our inclusion criteria, of which most were policy initiatives that banned certain types or uses of antimicrobials.

Antimicrobial use and AMR in production animals is a complex issue. Policymakers must preserve access to antimicrobials – an essential tool for animal welfare and production - while simultaneously working to ensure their usefulness is preserved for the future [5]. This complexity is further compounded by the sheer number of production animal systems that exist. Additionally, the relationships between stakeholders which influence antimicrobial use across these systems are convoluted: producers, industry groups, governments, veterinarian organizations, trade groups and consumer preferences can all influence policymakers' ability to regulate antimicrobial use. Finally, initiatives targeting antimicrobial use in animals are complicated by the fact that they must also account for animal welfare and economic impacts for producers and farmers who often operate on thin profit margins. Just as there is no perfect policy solution to address antimicrobial use and AMR across all cultural and socioeconomic settings, there is no policy solution applicable across all animal systems. Instead, a variety of policies targeting these systems are needed.

While the government policy initiatives that we identified in our review that have been implemented and evaluated thus far are useful, we argue that there is a need for more evidence-informed, diversified, and systematically evaluated AMR policy solutions to reduce antimicrobial use in animal systems.

Our systematic review identified interventions that can be broadly classified into two policy categories in the Behaviour Change Wheel framework [3]: regulatory interventions or guideline interventions. Most policies could be further classified into three typologies: those banning certain types (e.g., fluoroquinolones) or uses (e.g., growth promotion) of antimicrobials in production animals, or those implementing antimicrobial guidelines. By comparison, a 2019 systematic review of government policies targeting human antimicrobial use [6] identified a much wider variety of policy types, including those focused on improved infection prevention and control, changing prescriber practices, or educating the public.

There is no reason to believe these interventions would be ineffective in production animal settings. Similar approaches could be applied to target farm biosecurity, enhance veterinary education on antimicrobial stewardship, and provide direct training and outreach to farmers and producers. In fact, governments may be uniquely positioned to implement policies like mandated antimicrobial education across veterinary schools. Additionally, while some animal industry organizations may have already implemented some of these policies, we did not identify any that had partnered with governments. For example, a successful voluntary reduction in antimicrobial use was achieved by Chicken Farmers of Canada when they announced the poultry industry would no longer use category I or II antimicrobials, which are considered of higher importance for human health, in poultry feed or water for prophylaxis [7]. Industry-government partnerships which direct regulation may help drive faster uptake of these policies by producers.

While governments may have implemented a variety of policies targeting AMU or AMR in production animals [8], evaluations appear infrequently in peer-reviewed literature. Without exploring the impacts of these policies, we risk implementing ineffective or unsustainable interventions, which may even exacerbate the threat of AMR. Since our knowledge of the link between antimicrobial use and AMR in production animals is still tenuous [9], these evaluations can also supply key evidence needed to further our understanding of this causal link. The limited evaluation of these policies in the literature may also reflect the difficulty of publishing adverse or null findings, which are often seen as reflecting poorly on government initiatives, rather than being recognized as valuable evidence to inform and improve future policymaking.

We urge policymakers to think “outside the box” and consider the full breadth of policies that could apply to production animal systems going forward. Partnerships and dialogue with industry groups and producers will also help encourage faster uptake from producers and will increase the likelihood that the policies being proposed are feasible and effective. Concurrently, policymakers need to ensure that the policies they implement are evaluated so that they can be used to inform future initiatives.

## CRediT authorship contribution statement

**Fiona Emdin:** Writing – original draft, Conceptualization. **Kayla Strong:** Writing – review & editing, Conceptualization. **Daniela Corno:** Writing – review & editing, Conceptualization. **Jaskeerat Singh:** Writing – review & editing, Conceptualization. **Susan Rogers Van Katwyk:** Writing – review & editing, Project administration. **Heather Ganshorn:** Writing – review & editing, Methodology. **Arne Ruckert:** Writing – review & editing, Writing – original draft, Validation, Conceptualization. **Jeremy M. Grimshaw:** Writing – review & editing, Conceptualization. **Mathieu JP Poirier:** Writing – review & editing, Supervision, Funding acquisition, Conceptualization.

## Author statement

All authors contributed to the study conception and design. All authors read and approved the final manuscript.

## Funding

This work is supported by the 10.13039/501100000024Canadian Institutes of Health Research [#149542], the Social Sciences & Humanities Research Council [#895-2022-1015], and the 10.13039/100010269Wellcome Trust [222422/Z/21/Z]. The authors and the protocol development were independent of the funding organization. The funding bodies were not involved in the study design, data collection, analysis, interpretation, or writing.

## Declaration of competing interest

The authors declare no competing interests. This commentary is based on insights derived from a systematic review conducted by the authors. The purpose of this article is to critically reflect on the current state of evidence and offer policy-relevant recommendations to reduce veterinary antimicrobial consumption. The authors have no financial, personal, or professional affiliations that could be perceived to influence the content of this work.

## Data Availability

No new data were generated or analysed during this study. All data cited are from publicly available published sources.
